# Cryopreservation and Fertility: Current and Prospective Possibilities for Female Cancer Patients

**DOI:** 10.5402/2011/350813

**Published:** 2011-12-01

**Authors:** Jacira Ribeiro Campos, Ana Carolina Japur de Sá Rosa-e-Silva

**Affiliations:** Department of Obstetrics and Gynecology, Faculty of Medicine of Ribeirão Preto, University of São Paulo, 14049-900 Ribeirão Preto, SP, Brazil

## Abstract

With the evolution of the treatment of malignant neoplasms, the survival rates of patients undergoing chemo- or radiotherapy are increasing. The continuous development of techniques of assisted human reproduction has led to important strategies in an attempt to maintain reproductive function in patients subjected to treatment of neoplastic diseases, among them cryopreservation of embryos, gametes, and ovarian cortical tissue. The freezing of ovarian tissue is currently being proposed with the primary purpose of preserving ovarian function in these patients. Currently, the major challenge of groups working with preservation of fertility is the use of cryopreserved ovarian tissue after disease remission. The main alternatives presented today are the implantation of hetero- or orthotopic tissue and isolation of immature follicles from ovarian tissue followed by *in vitro* maturation and assisted reproduction procedures.

## 1. Introduction

Invasive cancer is diagnosed in more than 600,000 persons per year in the United States, with 269,800 of affected patients being women, 8% of whom are younger than 40 years [1]. With the use of modern protocols for the treatment of cancer, 80% of the patients will survive. It has been estimated that, over the next few years, one in each 250 young adult women will be a survivor of childhood cancer, with some side effects [2, 3]. One of the main problems, especially for young patients, is that therapy destroys a significant proportion of the follicular population, a fact that may result in permanent infertility [3–5].

Although treatment and survival are the main focus of both health professionals and cancer patients, it is important to consider the quality of life of the latter after treatment, including the possibility to have children [6].

The cytotoxic action of ionizing radiation and chemotherapeutic agents frequently leads to the occurrence of premature ovarian failure (POF), with serious long-term consequences such as reduction of bone mass leading to osteoporosis, to an increased incidence of cardiovascular diseases, and to the premature onset of climacteric and infertility symptoms [7]. The temporary or permanent absence of the endocrine and reproductive ovarian functions mainly depends on the age of the patient at the time of gonadotoxic treatment and on the doses administered. The risk of infertility varies according to treatment and to the type of chemotherapy based on alkylating agents and platinum, which have a greater potential to damage the reproductive system [6, 8, 9]. Several types of cancer treatment can cause infertility in patients looking for a cure. Some chemotherapy protocols can cause POF or acute ovarian insufficiency during or soon after cancer treatment.

The occurrence of POF is observed in 69% of patients aged up to 29 years and in 96% of patients aged 30 years or older after chemotherapy for the treatment of Hodgkin's disease [7]. For women submitted to radiotherapy with doses higher than 300 cGy, the incidence of POF can be higher than 60%, with the exposure to 4 Gy of pelvic irradiation being lethal for the oocytes, and the exposure to 5 to 10.5 Gy leading to ovarian failure in 97% of patients. The combination of chemotherapy and radiotherapy is particularly devastating. However, studies on women who conceived after chemotherapy did not detect a significant increase in congenital malformations or malignant neoplasias in the offspring [5, 10–12].

More and more patients are currently surviving cancer thanks to an early diagnosis and adequate treatment. Although surviving adults tend to be more interested in the preservation of fertility, it is important to remember that other options should be made available to young cancer patients. Health professionals should be prepared to discuss fertility with their patients regardless of the age or marital status of the latter at the time when therapy is started, so that they may understand the possible consequences of cancer treatment and the alternatives available for the preservation of fertility [13].

Other groups of patients may be candidates for the preservation of fertility, such as women with endometriosis and patients treated with chemotherapeutic agents for nonmalignant diseases such as autoimmune and collagen-vascular conditions. Persons with deficiencies in DNA repair are also more susceptible to gonadotoxicity [13, 14]. Other cases include the incompatibility between age of maternity and career plans or simply women who have not yet found the desired partner in order to start a family [15].

The continuous evolution of assisted human reproduction techniques has permitted several strategies to be considered for the maintenance of reproductive function in female patients who complete treatment for neoplastic diseases, such as hormone manipulation, ovary transposition, and cryopreservation of embryos, gametes, and ovarian cortical tissue.

## 2. Cryoprotectors and Freezing Methods

Cryopreservation consists of the preservation of biological material at low temperatures, usually in liquid nitrogen at −196°C, or in the vapor phase of nitrogen at −150°C. The only physical states existing below approximately −130°C are the crystalline and the vitreous ones. In both states, viscosity is quite high, diffusion is considered to be nonsignificant (depending on storage time), the molecular kinetic energy is very low, and metabolic reactions driven by thermal energy occur very slowly or are completely paralyzed. Thus, at the temperature of liquid nitrogen, viability during storage can be extended over long periods of time, with maintenance of the stability of genetic material. The ability of biological material to survive the cryopreservation process depends on its tolerance of cryoprotective agents, dehydration, cooling and reheating [16].

Cryoprotective agents are substances that protect cells against dehydration, cooling, and damage caused by extreme temperature reduction. In general, these agents can act by (1) penetrating the cells (intracellular cryoprotectors) and replacing the water molecules of the cell, (2) reducing the freezing point, (3) protecting the cell membranes (extracellular cryoprotectors) by binding to the heads of the phospholipid groups, (4) increasing the viscosity of the medium, or (5) reducing the electrolyte concentration during cryopreservation, thus reducing the risk of osmotic damage. However, the cryoprotective agents can be toxic and can also facilitate the entry of toxic agents into the cells [17].

The use of cryoprotectors reduces cell damage during tissue preservation, although cryoprotectors are indispensable for a successful process, cytotoxicity may occur in cases of prolonged exposure to these agents, with a l5–90% loss of primordial follicles occurring according to the protector used [18]. The cryoprotective agents may be alcohols, amines, sugars, and proteins, and their effectiveness depends on some properties such as high solubility in water in order to minimize the osmotic gradient and to reduce toxicity. The most common cryoprotective solutions consist of the following combinations: (a) dymethyl-sulfoxide and human serum, (b) 1.5 M ethylene glycol, 1.5 M dymethyl-sulfoxide and fetal calf serum, and (c) 1-2-propanediol, 0.1 M sucrose, and human serum albumin [19].

### 2.1. Slow Freezing

Slow freezing is characterized by cell or tissue exposure to low concentrations of the cryoprotective agent for a period of time ranging from 20 to 60 min [20–23]. With this method, the material is cooled slowly at the rate of 2°C/min to −4 to −9°C and is kept at this temperature for a short period of time (10 to 15 min) for thermal stabilization and seeding, which prevents supercooling and extreme cell dehydration. The sample then continues to be cooled slowly at the rate of 0.3°C/min. Once a sufficient cell dehydration is reached (−30 to −80°C), the material is stored in liquid nitrogen (−196°C). This technique has proved to be effective for the conservation of ovarian tissue [24, 25]. Its use requires specific high-cost equipment but, on the other hand, it requires lower cryoprotector concentrations, with less toxicity to the cells.

### 2.2. Vitrification

Vitrification was first conceived by Luyet in 1937 and almost 50 years later Rall and Fahy [26] described vitrification as an alternative to the process of slow freezing. In contrast to slow freezing, vitrification involves the exposure of biological material to high concentrations of the cryoprotective agent for a short period of time (25 seconds to 5 min) usually at room temperature, followed by ultrarapid cooling in liquid nitrogen, without the need to use sophisticated and highly expensive equipment. According to Stachecki and Cohen [27], vitrification involves two basic aspects to be taken into consideration; the first consists of the fact that the high concentrations of cryoprotective agents used for exposure increase the toxic effects, and the second is that, despite this effect during the period of equilibrium, vitrification, by involving very rapid freezing, increases the survival rates. Vitrification has been successfully used for ovarian tissue, with minimal changes in tissue morphology [28, 29].

Although both techniques are being contemplated for the clinical application of embryo, gamete, and ovarian tissue cryopreservation, the technique of choice for the procedure varies according to the type of material to be conserved, without full consensus among authors.

## 3. Clinical Applications of Embryo, Gamete, and Ovarian Tissue Cryopreservation

### 3.1. Embryo Cryopreservation

The only method established thus far and routinely used at assisted reproduction clinics is embryo cryopreservation. However, this option requires ovarian stimulation, oocyte collection, and the use of assisted reproduction techniques that may take 2 to 5 weeks. Thus, the delay of chemotherapy prior to embryo storage is not feasible for some patients and may even lead to a worse prognosis for some cancer types. Embryo freezing also does not apply to patients without a steady partner with whom they wish to produce offspring in the future, as is the case for adolescents. An alternative would be the use of semen from a bank, although this option involves religious, moral, and ethical questions [3, 8].

In addition, stimulation should not be performed in women with hormone-dependent tumors such as breast cancer although new stimulation protocols have arisen with the use tamoxifen and of aromatase inhibitors (Letrozole), showing encouraging results [30]. On this basis, embryo freezing should be considered as the first option for the preservation of fertility in women with a steady partner who are in a condition to be submitted to at least one *in vitro *fertilization (IVF) cycle and is currently the only technique officially recognized by the American Society of Clinical Oncology for the preservation of fertility [31]. Although this is the recommendation, it is important to emphasize that a successful preservation of fertility is not guaranteed in view of the limitation of the number of embryos that can be obtained and frozen in a stimulation cycle.

It should be remembered that the rates of live births after embryo freezing-thawing depend on patient age and on the number and quality of cryopreserved embryos. Based on these variables, the mean pregnancy rate after embryo freezing-thawing is about 20 to 30% with the transfer of 2 to 3 embryos and the rate of live born babies per embryo transfer cycle is 27.7%, as reported by the Center for Disease Control and Prevention [32] (2006 data). Although oocytes can be retrieved without ovarian stimulation (natural cycle), the final yield of embryo production in nonstimulated cycles is extremely low [31] and therefore not recommended for cancer patients [30].

Another point to be remembered concerns the ethical implications of embryo freezing. Since cancer is a disease with a potentially unfavorable outcome leading to patient death, couples should be informed about the lack of ethical or legal possibility of disposal of these embryos in case of death of one spouse. Furthermore, even if oncologic treatment is successful, embryo transfer to the uterus will occur only after several years of disease remission and, regardless of the situation of the relationship of a couple after these years, the embryos exist and need a destination either to the maternal uterus or to donation for research.

Some ethical questions should be raised regarding the decision to be made by the couple: if the maternal partner dies, what is the right of the male partner (father) to these embryos? In this case, would it be possible to transfer these embryos to a new female partner of this biological father? How would the family of the deceased biological mother fit within this context? The Brazilian legislation has no replies to these questions, and there is no law regarding the destination of frozen embryos. All current conducts are based on the judgment of the team that assists the patient, on good counseling of the couple, and on the recommendations of the Federal Council of Medicine.

### 3.2. Cryopreservation of Mature Oocytes

Oocyte cryopreservation obviates many of these ethical questions associated with embryo cryopreservation. This technique has arisen as an alternative for the preservation of fertility and has the great advantage of being able to be used by women without a defined sex partner. However, a cycle of ovarian stimulation is required before the beginning of chemotherapy. Although the first birth obtained from frozen oocytes was reported by Chen in 1986, the results obtained at the time using IVF of thawed oocytes were poor, with low rates of oocyte survival, fertilization, and pregnancy [14]. However, starting in 2005, with the use of oocyte vitrification techniques there was an increase in the rate of survival of frozen-thawed oocytes and also of pregnancy [33]. Kuwayama et al. [34] developed as specific system for specimen storage and revolutionized the results of oocyte freezing using the vitrification technique. In 2005, the authors obtained a 91% rate of oocyte survival after freezing-thawing, an 81% cleavage rate, a 50% rate of blastocyst formation, and a 41% pregnancy rate after embryo transfer. Antinori et al. [35] obtained a 99.4% rate of oocyte survival, with fecundation, pregnancy, and implantation rates of 92.9, 32.5, and 13.2%, respectively, and Cobo et al. [36] obtained a 96.6% rate of oocyte survival, with a 65% pregnancy rate. These results are similar to those reported for fresh oocytes and indicate a revolution in the techniques of gamete cryopreservation. Despite these results, the freezing of mature oocytes is still considered to be experimental by the American Society of Clinical Oncology [31].

### 3.3. Cryopreservation of Immature Oocytes

This method emerged as an alternative to the freezing of mature oocytes since immature oocytes are believed to be more resistant to the freezing process by being more undifferentiated, by the absence of a spindle and by having chromosomes protected by a nuclear membrane. Some authors propose the puncture of still immature oocytes and the application of *in vitro* maturation (IVM) techniques associated with the cryopreservation of these gametes in order to minimize the time of induction and reduce the costs and side effects of controlled ovarian hyperstimulation [30, 37]. However, results regarding oocyte survival and maturation up to metaphase II do not seem to be very encouraging. This method will not be valid until IVM of oocytes becomes a routine procedure [38].

### 3.4. Cryopreservation of Cortical Ovarian Tissue

The cryopreservation of ovarian tissue is based on the principle that inactive (primordial) follicles resist cytotoxicity better than follicles in the process of maturation [39] since their metabolism is reduced, the zona pellucida is absent, and the cryoprotectors can penetrate more easily due to the smaller follicular size [40]. In addition, primordial follicles have a greater potential for the repair of damage suffered by the organelles and by other structures during the prolonged freezing phase in cryopreservation.

The functional cell damage caused by cryopreservation, however, can be irreversible if the temperature falls below ideal levels. Very abrupt thermal variations may interfere with water transport through the cell membrane and favor the formation of ice crystals and salt deposits inside the cell [41]. The differences in osmotic pressure between the intra- and extracellular media may also lead to a change in oocyte volume, with consequent damage to the plasma membrane and to the organelles [42]. Another factor that may favor cell damage is the presence of meiotic spindles in the follicular cells. It has been demonstrated that small variations in temperature can damage the microtubules that compose the spindle, possibly leading to chromosome losses and aneuploidies during the end of the first meiotic division in the subsequent maturation process. In addition, it has also been demonstrated that freezing can damage the cytoskeleton of the cell or the oocytes, with impaired transit of molecules and organelles during the process of cell division. The phase of tissue reexpansion (thawing) can also be deleterious if the external medium is not adequate [43].

The freezing of ovarian tissues is being currently proposed mainly in order to maintain preserved ovarian function in terms of both fertility and hormone production, a goal that is not reached with the remaining methods. In addition, this option currently emerges to serve specific groups of patients for whom the remaining techniques are not recommended, such as (1) prepubertal patients whose gonads are not yet under the control of the hypothalamus-pituitary axis, (2) women without a partner who do not desire embryos obtained by fertilization with donor semen, (3) patients with estrogen-dependent neoplasias such as breast cancer, and (4) women with malignant neoplasias which require an immediate approach, for whom a delayed onset of treatment for the time needed to induce ovulation may lead to changes in prognosis. Particularly regarding this aspect, the collection of cortical ovarian tissue for cryopreservation has the advantage of being possible to perform at any time during the menstrual cycle of the patient and of permitting the acquisition of hundreds of primordial follicles. The disadvantages of the cryopreservation of cortical ovarian tissue deserve some comment and consist of (1) submitting the patient to surgical procedures for the collection of ovarian tissue and (2) the possibility of reintroducing malignant cells at the time of later tissue reimplantation [44, 45].

The procedures of cryopreservation should guarantee follicular viability and tissue integrity in cell-to-cell contact. Today, the slow cooling method is being essentially used in clinical programs. Vitrification of the ovarian cortex, a supplementary technique which is currently being developed, has also elicited considerable interest [46].

In the ovaries of adult mammals, the follicles are present in various stages of development. Primordial follicles can be considered to the “storage form” of oocytes in the ovary, representing a valuable source of oocytes that could be used for fertility purposes [47, 48]. Although many authors believe that freezing ovarian tissue is a promising technique for the preservation of fertility, damage to the follicular nucleus and DNA of sheep and humans after freezing-thawing has been reported in studies using immunohistochemistry, fluorescent *in situ* hybridization, and final labeling of the deoxynucleotidyl transferase terminal [12, 49].

The process of ovary freezing and thawing can cause tissue damage, possibly due to the formation of intracellular ice and to the toxicity of the cryopotectors [50]. Once the ovary is cryopreserved, the major challenge of this technique as a method for the preservation of fertility is the later use of thawed tissue. Among the possibilities considered, there is the reimplantation of tissue as a whole or as fragments [51] or the isolation of immature follicles by a digestion technique or by microdissection, followed by IVM of primordial and primary follicles [52]. It should be pointed out that many researchers have invested in IVM of primordial follicles.

Regarding reimplantation of thawed ovarian tissue, the major obstacles in the way of successful restoration of fertility with frozen-thawed ovarian cortex are adherences and massive ischemic damage to the follicles until the development of neovascularization. Most of the follicles that survive cryopreservation suffer ischemia during the time needed for neorevascularization [12].

Cortical ovarian tissue can be collected by laparoscopy or laparotomy by obtaining various biopsies, or by total or partial uni-or bilateral oophorectomy followed by cryopreservation of the tissue [45]. The protocols most extensively used to freeze ovarian tissue involve techniques of slow progressive (ramp) freezing [24] and vitrification in liquid nitrogen [34].

Efforts are currently directed at the attempt to elucidate the potential of frozen-thawed ovarian tissue for the maintenance of reproductive capacity using either *in vitro* maturation of isolated oocytes or autotransplantation of cryopreserved tissue [52].

#### 3.4.1. Tissue Reimplantation after Freezing and Thawing

Several studies were conducted in the fifties regarding ovary cryopreservation and transplantation. Gosden et al. [24] in 1994 obtained successful restoration of fertility in sheep after autologous transplantation of frozen-thawed ovarian tissue and showed new perspectives for the technique, especially as a strategy to preserve ovarian function in women with cancer.

Frozen-thawed ovarian tissue can be transplanted in two ways: orthotopic transplant—the ovary or ovary fragment is placed in the ovarian fossa close to the infundibulo pelvic ligament, and heterothotopic transplant—the tissue is implanted at any site outside its original *locus*. A spontaneous pregnancy can be theoretically obtained in a patient with pervious tubes. When the heterotopic transplant is used, pregnancy will only be possible using assisted reproduction procedures [53]. Considerable success has been achieved in terms of restoration of hormonal cyclicity in patients and, currently, 15 healthy children have been born worldwide as a result of transplanting frozen/thawed ovarian tissue [52].

#### 3.4.2. In Vitro Maturation (IVM)

The current great challenge for groups working with fertility preservation is the utilization of cryopreserved ovarian tissue after disease remission. As mentioned earlier, there are two major alternatives: reimplantation of hetero- or orthotopic tissue and isolation of immature follicles from ovarian tissue followed by IVM and assisted reproduction procedures. The first option, in addition to involving technical difficulties related to graft ischemia, also involves the possibility of reimplantation of tumor cells together with tissue. On this basis, IVM becomes an option of high clinical relevance in which much research has been invested.

The maturation of mammalian oocytes is a complex process involving the resumption and progression of the meiotic cycle and the reprogramming of cytoplasmic events, prerequisites for monosperm fertilization and early embryo development [52].

*In vivo*, the occurrence of oocyte maturation is characterized by the high developmental potential of the cell after fertilization. However, under experimental conditions (IVM or superovulation techniques), the quality of the oocyte is drastically reduced. This limiting step in the technology of *in vitro* embryo production has led to the formulation of different IVM methods based on culture media with cells or tissues added and/or supplemented with biological fluids in an attempt to mimic the events that occur in the follicular microenvironment [54–61].

The current scientific production about IVM mainly involves the maturation of immature oocytes already in the process of development (germinal vesicle). However, follicles isolated from ovarian tissue are still in a primordial and primary stage. The literature about these follicles is quite scarce, although several investigations have been conducted in order to improve the rate of *in vitro *maturation of these oocytes [62].

## 4. Final Considerations

Fertility preservation continues to advance with innovative investigations carried out in order to obviate the damage caused by oncologic treatment or even to help women who opt for a late pregnancy. Each method has advantages and limitations and all procedures involve social, ethical, and legal considerations. The techniques available to women who try to preserve fertility are embryo cryopreservation, oocyte cryopreservation, and ovarian tissue cryopreservation. Today, embryo cryopreservation is the official successful method of fertility preservation although its use is limited to women who have husbands or male partners or male partners who are willing to use sperm cryopreservation. The techniques of ovarian tissue or oocyte cryopreservation are still considered to be experimental. These methods will permit girls and young women to become parents in the future even after exposure to chemotherapy or to other agents that might cause infertility. The prevention of reproductive failure would be an ideal approach and several studies on this topic are currently underway.
